# Nature and Treatment of Hæmoptysis

**Published:** 1844-07

**Authors:** 


*Nature and Treatment Hæmoptysis.*—Dr. Graves has very clearly explained the difference in hæmoptysis, when the blood comes from the bronchial or from the pulmonary arteries. The blood going to the lungs along the bronchial arteries being red, while that in the pulmonary is dark venous, it will be evident by the kind of blood which is expectorated from whence it originates. Dr. Graves has shown that when it arises from the branches of the pulmonary artery, it is in consequence of the direct effusion of blood from those branches which ramify on the air-cells, and that the blood expectorated has nothing to do with the bronchial mucous membrane or bronchial arteries; and that the blood may escape into the inter-vesicular pulmonary tissue, where, having no exit like the portion which is thrown out into the air-cells, it may remain. This constitutes pulmonary apoplexy. He does not allow that when blood expectorated is dark venous, it necessarily comes from the stomach, but correctly shows that it may be equally dark and venous when arising from these pulmonary branches; and that when the stethescope is applied, the signs of disease of the lungs will often be evident enough; at the same time even blood from these vessels may be rather red, owing to its partial aëration. From these circumstances, when we find that in pneumonia the expectoration is tinged with dark venous instead of arterial blood, we may conclude that it is of a more dangerous nature than when the bronchial vessels only are implicated. When these last mentioned arteries give rise to the hemorrhage, it is generally of a less dangerous nature and more scanty; although it occasionally happens that even when from this source it becomes alarming. One very important indication of treatment in cases of hæmoptysis is to relieve the congestion and arrest the further effusion of blood, by copious depletion; and the extent and promptitude of this measure will depend upon the fact whether the hemorrhage is arising from the pulmonary or from the bronchial vessels. If from the former it will be more dangerous and extensive, owing to the blood plugging up and obliterating the ultimate divisions of the air-tubes, perhaps to a great extent of surface; but if from the bronchial vessels only, this effusion will seldom be very extensive, and not nearly so dangerous as in the former instance, arising in phthisical cases perhaps rather from the congested state of the bronchial membrane than from ulcerative action implicating the vascular tissue. When free depletion has been adopted, according to the case, Dr. Graves relies chiefly on ipecacuanha given in doses of two grains every quarter of an hour, until there is some improvement, and then every half hour or hour until the bleeding stops. This remedy is to be preceded by a purgative enema and a saline cathartic. The ipecacuanha is recommended not only in these cases, but also in hemorrhage from the bowels, and even in hæmatemesis in preference to the acetate of lead, which Dr. Graves employs only in those cases of passive hemorrhage in which opium is indicated, and then in combination with the last remedy. We have tried this treatment with great success in our own practice, but have been equally successful with the tartarized antimony, except in those cases where the alimentary canal has been the part affected; and we suspect that it will be found that the same good effect wlll arise from all or most medicines which have a nauseating influence on the system.—*Ibid.*

